# Biodiversity science blossoms in China

**DOI:** 10.1093/nsr/nwab058

**Published:** 2021-06-05

**Authors:** Richard B Primack

**Affiliations:** Biology Department, Boston University, USA

Over the past 35 years, China has been transformed by an economic miracle unlike anything seen in the history of the world. Hundreds of millions of people have emerged from rural poverty, cities have been re-built, cutting-edge industries have been established and a modern transportation network now knits together the country. This transformation has come at a significant cost to the environment, in terms of air pollution, water pollution, the loss and fragmentation of habitats, and threats of species extinction. Yet, as the review by Mi *et al.* (2021) points out, China is also now emerging as a leader in biodiversity conservation and research [1]. This focus on finding a balance between biodiversity conservation and socio-economic development, sometimes referred to as ‘ecological civilization,’ is a high priority in China because of the dependence of its enormous human population on ecosystem services and because of its astonishing richness of species.

As anyone active in this field can readily observe, the number of researchers and research projects, and the amount of funding in ecology, conservation, wildlife biology and evolutionary biology are growing more rapidly in China than in any other place in the world. Ecological and conservation projects are often being launched on a larger scale and with more staff and resources than similar projects in North America and Europe. In many of these initiatives, early- and mid-career Chinese scientists, who have often studied abroad on fellowships and sabbaticals, work collaboratively with outstanding foreign scientists who hold positions in Chinese universities and institutes. Despite this growth in projects and staffing, women and other groups are underrepresented; in 2017, just 6% of the members of the Chinese Academy of Sciences were women [2]. Increasing participation of women and other groups could further broaden the diversity of ideas and increase equity in conservation research.

Over the past three decades, universities across China have been dramatically upgraded, in terms of the physical infrastructure and the quality of the academic staff and student experience. Their aim has been to achieve on-the-ground, results-oriented, educational programs and to conduct them with greater academic rigor and ethics. Whereas in North America and Europe, scientists often work on local or regional scales and on individual projects, China's strong centralized funding and political structure allows more effective national-scale projects. For example, Mi *et al.* (2021) report that the Chinese Academy of Sciences organized 850 universities to assess the natural resources and biodiversity of China [1]. By focusing its scientists on selected priority topics, the Chinese scientific establishment has been able to tackle important and comprehensive projects, such as the massive *Flora of China* initiative with over 30 000 plant species covered in 80 volumes. Similarly, 160 volumes of the *Fauna of China* have been published so far. The treatment of life in the South China seas includes over 28 000 marine species. These examples illustrate the ability of the scientific establishment to harness the people and resources of China to complete ambitious projects.

Mi *et al.* (2021) also describe many other national-level initiatives such as the Red List evaluation of species, redline policies to protect ecosystem services and biodiversity, canopy cranes, remote sensing, plant phenology networks, gene sequencing and the network of forest dynamics plots, all carried out by large teams of researchers at multiple locations [1]. The scale of Chinese investment in biodiversity research is illustrated by two specific projects:

1. In the past, biologists in China and elsewhere were often severely limited in their ability to access museum specimens, which were held in scattered locations around a country or around the world. China has been a leader in the international effort to digitize museum collections and make images available online [3]. China has already digitized 15.7 million herbarium specimens from 329 herbaria. By comparison, Germany has digitized about 3 million specimens, and California perhaps a few million specimens. In addition, China has digitized 13 million color photographs, which have the same value as museum specimens for many research purposes. This digitization effort has greatly increased the ability of scientists anywhere in the world to carry out ecological studies related to climate change, plant distribution and conservation.
2. China is also leading international efforts to establish experiments and monitoring networks to determine how biodiversity and species richness contribute to ecosystem services, such as carbon storage, nutrient uptake and biomass production [4]. Chinese scientists have established numerous long-term studies and experiments in grasslands on the Qinghai-Tibetan plateau and in Inner Mongolia [5,6]. These studies contribute to a larger network of 6098 forest, shrubland and grassland plots across China where researchers are monitoring biodiversity and ecosystem processes. China's unique capacity for mega-experiments is exemplified by whole forest-scale biodiversity experiments, in which researchers established entire forests with different numbers of tree species in different plots within the forests, with up to 42 tree species in the most
3. diverse plots. It is hard to imagine another country with the resources and staff to carry out such ambitious, large-scale experiments.

**Figure 1.**
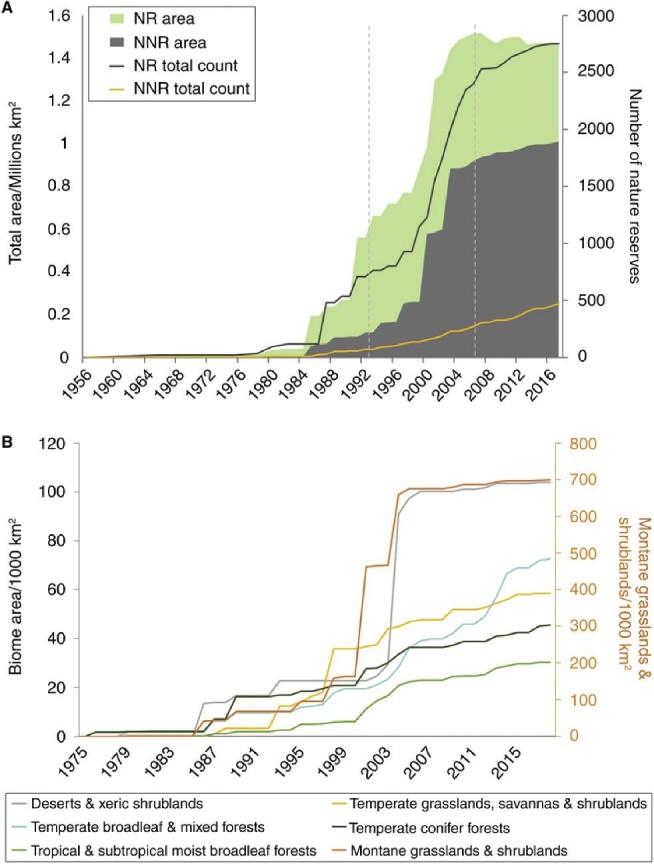
Growth in the area of national nature reserves (green) and local nature reserves in China (gray) from the 1950s to the present. Number of reserves are shown as lines. From Li and Pimm, 2020 [10].

Between 1980 and 2005, China expanded the number of national nature reserves (similar to national parks elsewhere) from virtually none to over 400 parks protecting close to 100 million hectares (Fig. 1) [7]. China was able to use the wealth generated by its rapid economic growth to fund the dramatic expansion of protected areas and to provide alternative jobs and living situations for people impacted by the creation of new protected areas. The government also had the political will to expand protected areas and deal with any concerns being raised [8]. Since 2005, the area of nature reserves in China appears to have stabilized or even declined as economic pressures prevented additional land from being protected, and even led to many existing parks being degazetted. Research also shows that the protected areas are mismatched with the distribution of biodiversity and ecosystem services—they do not protect nearly as much biodiversity or threatened species as they could [9]. Despite these concerns, there has been consistent progress towards the protection of forests and forest species [10]. In addition, smaller nature reserves are being aggregated into larger national park units, which can be managed more effectively [11].

This comprehensive review by Mi *et al.* (2021) also highlights the dilemma facing biodiversity scientists and conservation researchers working in China today [1]. While China's capacity to investigate its astonishing biodiversity is increasing, the country's rapidly expanding economy continues to threaten that biodiversity. Will China reach an equilibrium at which its remaining biodiversity is protected and economic development stabilizes? Or will the economy keep expanding, leading to the destruction, fragmentation and degradation of ecosystems and the extinction of large numbers of species? We can only hope that China's growth in conservation research helps the country achieve the former ecological civilization and avoid the latter disaster.

***Conflict of interest statement.*** None declared.
